# Open triple-branched stent graft applied to patient of acute type a aortic dissection with Aberrant Right Subclavian Artery

**DOI:** 10.1186/1749-8090-8-85

**Published:** 2013-04-15

**Authors:** Changfa Guo, Kai Zhu, Demin Xu, Chunsheng Wang

**Affiliations:** 1 Department of Cardiac Surgery, Zhongshan Hospital, Fudan University, No. 180, Fenglin Road, Xuhui District, Shanghai, 200032, P. R. China; 2 Shanghai Institute of Cardiovascular Diseases, No. 180, Fenglin Road, Xuhui District, Shanghai, 200032, P. R. China

**Keywords:** Aberrant right subclavian artery, Standford type A aortic dissection, Triple-branched stent graft

## Abstract

A 57-year-old Chinese male patient presented with Standford type A aortic dissection with an aberrant right subclavian artery (ARSA). At operation, the ascending aorta was replaced by a mono–branch vascular prosthesis with the branch bypassing to the ARSA; the triple-branched stent graft was inserted into the true lumen of the arch and proximal descending aorta (covering the origin of the ARSA) with each sidearm graft being positioned into the aortic branches; and then its proximal end was sutured to mono–branched vascular prosthesis. Follow-up computed tomography angiography showed false lumen of the dissection disappeared with satisfactory position of the triple-branched stent graft.

## Background

Acute aortic type A dissection is a life-threatening disease. The disease is very rare when it is combined with an aberrant right subclavian artery (ARSA), and the surgical treatment is difficult. In our present study, we used a novel triple-branched stent graft technique combined with mono-branched vascular prosthesis replacement to treat the disease, and the treatment was successful.

## Case report

**Figure 1:**
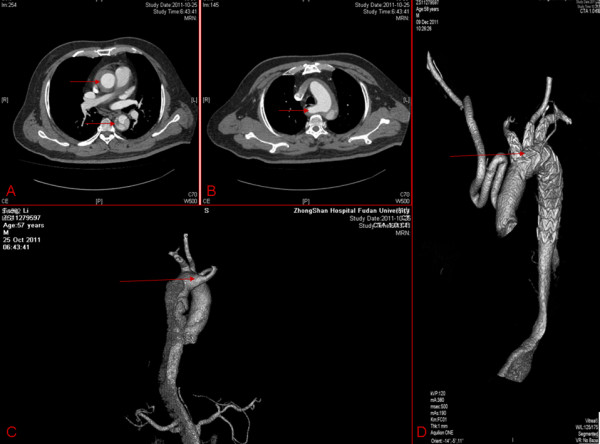
**The patient's CTA before and after the operation. A**: Standford type A aortic dissection; **B**, **C**: aberrant right subclavian artery; **D**: good position of triple-branched stent graft after operation.

A 57-year-old Chinese man with a history of severe hypertension for 10 years presented an acute severe chest pain with back irradiation. Echocardiography revealed an acute Standford type A aortic dissection with a dilated ascending aortic measuring 5.0 cm in diameter. Computed tomography angiography (CTA) confirmed the diagnose of Standford type A aortic dissection (Figure 1A) with ARSA (Figure 1B, C).

At operation, standard cannulation of the right atrium and right common femoral artery were carried out. The ascending aorta was replaced by a mono–branched vascular prosthesis with the branch bypassing to the ARSA. When the circulation was arrested with the temperature of 20°C, through the transverse incision of the ascending aorta, we found the site of primary entry originating from the distal portion of the aortic arch, just near the origin of the aberrant right subclavian artery. Then the triple-branched stent graft was inserted into the true lumen of the arch and proximal descending aorta (covering the origin of the ARSA) with each sidearm graft being positioned one by one into the aortic branches. Then, its proximal end was sutured to mono–branched vascular prosthesis. The cardiopulmonary bypass time was 249 minutes with aortic cross-clamp time of 72 minutes and circulation arrest time of 12 minutes.

The patient recovered well, and after one month, the follow-up CTA showed false lumen of the dissection disappeared with good position of the triple-branched stent graft (Figure 1D). This case demonstrated the first successful treatment of acute Standford type A aortic dissection with ARSA using a novel triple-branched stent graft technique.

## Discussion

Aberrant subclavian right artery (ARSA) is one common congenital aortic arch anomalies, occurring in approximately 0.5% to 1% of the population. It is thought to be persistence of the embryonic right dorsal aorta combined with involution of the arch segment between the right common carotid artery and right subclavian artery. And ARSA is most often asymptomatic unless a compression effect or atheromatous disease involvement occurs [1].

The acute angle of the ARSA may cause a weakening of the aortic wall, which could increase the risk of catastrophic aortic dissection [2]. And the type of aortic dissection depends on the orifice location of ARSA on the aortic arch. In previous reports, internal medicine, endovascular repair or surgical operation were performed respectively for patients with Standford type B aortic dissection with ARSA [3]. However, when Standford type A aortic dissection with ARSA was diagnosed, emergency surgical operation was indeed mandatory and risky. In traditional surgical operations, quadruple-branched grafts with elephant trunk were applied to do the total aortic arch replacement (all four arch vessels including ARSA were reconstructed individually using four branches), with the elephant trunk commonly inserting into the true lumen of proximal descending aorta to repair the dissection and cover the orifice of ARSA [2,4,5].

In our case, we utilized a triple-branched stent graft to propose the total arch replacement and covered the orifice of ARSA. The triple-branched stent graft consisted of a self-expandable nitinol stent and polyester vascular graft fabric (Yuhengjia Sci Tech Corp Ltd, Beijing, China). It comprised a main graft and 3 sidearm grafts. The main graft was tapered and flexible enough to conform to the curved aortic arch. According to the diameter of the patient’s vessels, we chose the stent graft with main graft of 26 mm and the 3 sidearm grafts of 14, 12, and 12 mm in diameter respectively. According to the previous experience, placement of these triple-branched stent grafts into the descending aorta, arch, and 3 arch vessels could be easily finished in 3 to 6 minutes during the procedure [6-8]. In our case, circulation arrest time was 12 minutes, which is much shorter than that used by the traditional surgical technique (total aortic arch replacement). It is no doubt that this shorter circulation arrest time will lead to an uneventful postoperative course, a short time on postoperative mechanical ventilation and a short stay in the intensive care unit. Further, our novel strategy could reduce the number of anastomotic stoma and avoid the isolation, blocking and anastomosis of aortic branches, which may increase the risk of bleeding and nerve damage. And as compared to traditional surgical technique (total aortic arch replacement), this technique is easier and safer with satisfactory results [6-8].

## Conclusions

In conclusion, we consider triple-branched stent graft associated with mono-branched vascular prosthesis mediated aortic arch replacement and ARSA reconstruction will enable a novel strategy for acute aortic dissection involving ARSA.

## Consent

Written informed consent was obtained from the next of the kin of the patient involved for publication of this case report and any accompanying images. A copy of the written consent is available for review by the Editor-in-Chief of this journal.

## Abbreviations

ARSA: Aberrant right subclavian artery; CTA: Computed tomography angiography.

## Competing interests

The authors declare that they have no competing interests.

## Authors’ contributions

CSW did the operations, conceived of the study, and helped in revising the manuscript critically. CFG and KZ participated in its design and coordination, and drafted the manuscript. DMX helped to revise the manuscript. All authors read and approved the final manuscript.
